# Meta-Analyses of Developing Brain Function in High-Risk and Emerged Bipolar Disorder

**DOI:** 10.3389/fpsyt.2014.00141

**Published:** 2014-11-03

**Authors:** Moon-Soo Lee, Purnima Anumagalla, Prasanth Talluri, Mani N. Pavuluri

**Affiliations:** ^1^Pediatric Brain Research and Intervention Center, University of Illinois at Chicago, Chicago, IL, USA; ^2^College of Medicine, Korea University, Seoul, South Korea

**Keywords:** pediatric bipolar disorder, high risk, meta-analysis, GingerALE, dorsolateral prefrontal cortex, amygdala

## Abstract

**Objectives:** Identifying early markers of brain function among those at high risk (HR) for pediatric bipolar disorder (PBD) could serve as a screening measure when children and adolescents present with subsyndromal clinical symptoms prior to the conversion to bipolar disorder. Studies on the offspring of patients with bipolar disorder who are genetically at HR have each been limited in establishing a biomarker, while an analytic review in summarizing the findings offers an improvised opportunity toward that goal.

**Methods:** An activation likelihood estimation (ALE) meta-analysis of mixed cognitive and emotional activities using the GingerALE software from the BrainMap Project was completed. The meta-analysis of all fMRI studies contained a total of 29 reports and included PBD, HR, and typically developing (TD) groups.

**Results:** The HR group showed significantly greater activation relative to the TD group in the right DLPFC–insular–parietal–cerebellar regions. Similarly, the HR group exhibited greater activity in the right DLPFC and insula as well as the left cerebellum compared to patients with PBD. Patients with PBD, relative to TD, showed greater activation in regions of the right amygdala, parahippocampal gyrus, medial PFC, left ventral striatum, and cerebellum and lower activation in the right VLPFC and the DLPFC.

**Conclusion:** The HR population showed increased activity, presumably indicating greater compensatory deployment, in relation to both the TD and the PBD, in the key cognition and emotion-processing regions, such as the DLPFC, insula, and parietal cortex. In contrast, patients with PBD, relative to HR and TD, showed decreased activity, which could indicate a decreased effort in multiple PFC regions in addition to widespread subcortical abnormalities, which are suggestive of a more entrenched disease process.

## Introduction

The relationship between pediatric and adult bipolar disorder has been the subject of controversy. It is not clear whether pediatric bipolar disorder (PBD) is the pediatric form of the typical adult bipolar disorder or an entity of its own, as bipolar disorder usually manifests differently in childhood than in adulthood. Some studies in adults have reported that a portion of adults with bipolar I disorder experienced childhood or adolescent onset, and some of them began showing symptoms even before 12 years of age (1, 2). Identifying early markers of brain function among those at high risk (HR) for PBD could serve as a screening measure when children and adolescents present with subsyndromal clinical symptoms prior to the conversion to bipolar disorder (pediatric or adult form). These biomarkers can also aid as a stand-alone bio-signature for the identification of risk even prior to the emergence of any clinical symptoms and could allow an opportunity to prevent the onset of full-blown illness (3). One way to begin identifying the biomarkers is to examine the brain function in the genetically HR offspring of patients with bipolar disorder. While some studies of HR have been published (4–11), due to their small sample sizes and corrections for multiple comparisons, the findings remain inconclusive.

To offer robust and reliable findings, we used a recently developed activation likelihood estimation (ALE) technique. This method assumes that the peak co-ordinates reported by each study represent the activation maps from which they are derived and uses the reported co-ordinates in voxel-wise analysis to assess the consistency of activation in any given set of studies (12–14). By performing the quantitative voxel-wise meta-analysis of already published results from the HR population and comparing them with those from the converted PBD and typically developing (TD) youth, we can provide objective, unbiased, and statistically based quantified evidence.

Ideally, a separate meta-analysis would be conducted for each individual domain, such as emotion processing or attention, as they relate to bipolar disorder diathesis. However, given the infancy of the current literature regarding HR patients, this is not practical, as no individual construct has included a sufficient number of studies to date. Instead, it is more feasible to study the commonalities probed across multiple domains in a systematic and statistically driven fashion. There is a certain advantage to combining all the studies that include multi-domain probes. First, the brain does not work in isolation across individual domains; therefore, it is necessary to examine the brain’s function as a whole while it is engaged in affective, cognitive, and motor control tasks (15). Furthermore, pooling several pilot studies produces an exploratory power of how the brain functions in a larger sample, eventually offering the possibility of correlating the results with the clinical manifestations of domains and disorders presenting with combined affective, cognitive, and motoric symptoms (16). This approach is a segue into future studies that can explore the interface of multiple domain functions in individual studies.

We consider emotional systems and circuits, in illness or wellness, to be closely linked to cognitive and motor control circuits of attention, working memory, and response inhibition (17). These systems interface at three tiers as shown in animal (18) and human studies of PBD (19): (1) at the prefrontal level between the ventrolateral prefrontal cortex [VLPFC; inferior frontal gyrus; Brodmann areas (BAs) 45, 47] and the dorsolateral prefrontal cortex (DLPFC; middle frontal gyrus; BAs 9, 9, 46), (2) at the intermediary cortex in the anterior cingulate cortex (ACC), such as between the dorsal (BA 32) and pregenual ACC (BA 24), and (3) at the subcortical level between the amygdala and striatum (19). While we could not determine which probe or domain dysfunction would contribute to activity in any given co-ordinate in this meta-analysis, we developed our hypotheses based on knowledge derived from the emerging literature. Emotion-processing tasks probing the affective systems entered into our meta-analysis would contribute to the increased prefrontal activity at the interface of VLPFC and DLPFC in HR and the decreased activity in PBD relative to TD (19). Increased subcortical amygdala activity would be a specific marker of PBD (20) relative to HR and TD. Based on our knowledge of attention and working memory task response, the DLPFC will manifest with increased activity in HR (6) and decreased activity in PBD (21), relative to TD. Impaired subcortical striatal activity would be a more entrenched specific marker of PBD’s cognitive and motor dysfunction (20, 22, 23) relative to the HR and TD groups.

## Materials and Methods

### Search strategy

**Figure 1:**
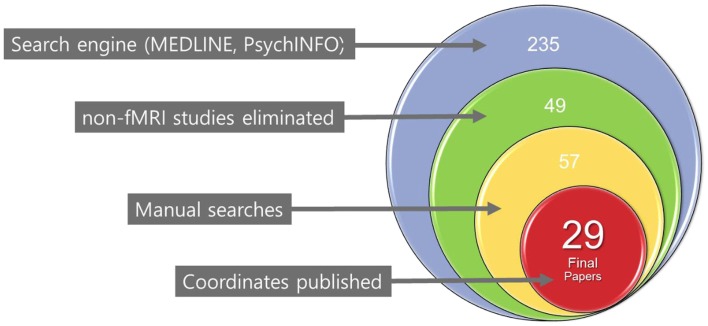
**Flow chart of the literature search for included studies**.

We identified primary studies through a comprehensive literature search of the MEDLINE (using both free-text and MeSH search) and PsychINFO databases using the following keywords: pediatric or child or adolescent, plus bipolar disorder or high-risk or at risk, and plus functional magnetic resonance imaging or fMRI. In addition, manual searches were conducted via reference sections of review articles and individual studies to check for any missing studies that were not identified using computerized searches. There were no language restrictions; in fact, all the included manuscripts were written in English. Only fMRI studies were chosen for review. An initial list of studies was produced that included any report of fMRI studies of PBD and HR offspring published in print or online by December 31, 2013. The selection process for the final list of primary studies for the planned meta-analyses in this study was very specific. The first-level literature search yielded 235 unique published articles with 49 studies meeting the initial inclusion criteria. A further manual search leads to eight other studies. After a second-level review of these 57 studies, only 29 contained the co-ordinates essential for inclusion in our meta-analysis (Figure 1). Any ambiguity in inclusion was resolved through a consensus decision by the authors of this manuscript. Study data (e.g., co-ordinates, participant numbers, and imaging spaces) were entered and crosschecked by participating authors.

### Selection criteria

“High risk” in this project refers to adolescents who have a biological parent diagnosed with BD. We selected studies with participants whose mean age was less than 19 years. Every study that we included had participants between the ages of 7 and 18 except for the study performed by Thermenos et al. (11), where the ages ranged up to 24. All reports included in the meta-analysis satisfied the following criteria: (1) a healthy comparison group is included, (2) the studies conducted whole-brain analyses, (3) all studies provided standard Talairach or Montreal Neurological Institute (MNI) spatial co-ordinates for the key findings, (4) patient participants had been diagnosed with bipolar disorder, and (5) there were at least five members in each of the participant groups. We included only those studies that reported activation foci as 3D co-ordinates in stereotactic space, examined active task constructs, and presented results for groups of participants.

Excluded manuscripts consisted of the following: (1) reviews or meta-analyses, (2) those with subject overlap, and (3) other MRI modalities (e.g., structural imaging, spectroscopy, diffusion tensor imaging, and functional connectivity studies).

### Activation likelihood estimation methods and pairwise ALE meta-analysis

GingerALE software version (version 2.3.1) from the BrainMap project was used to conduct ALE meta-analysis of eligible studies (13, 14, 43). Meta-analyses were performed using the revised ALE software (i.e., GingerALE 2.3). The key modification in the revised ALE software included the change from fixed-effects (convergence between foci) to random-effects inference (convergence between studies but not individual foci reported for the same study), as well as greater meta-analytic weighting for primary studies that involved more participants. In line with our goal of gaining insight on the whole brain’s function through tasks that probe combined domains, we performed exploratory analyses using all eligible data in the HR offspring, BD patient, and TD groups in the pediatric age group. Conversely, we did not separate the analyses by the type of the task or the brain domain probed. This method also helped to harness sample size and power. Activation co-ordinates reported in the MNI space were converted to Talairach co-ordinates using the Lancaster transform (icbm2tal) in GingerALE. Our meta-analysis was conducted in Talairach space. Co-ordinates originally presented as MNI space were transformed into Talairach space using Lancaster transformation. For uniformity, Talairach co-ordinates expressed by the previous Brett transformation (44) were converted into MNI space and re-transformed into Talairach space. The meta-analysis was performed using pairwise ALE meta-analysis.

Pairwise ALE meta-analyses included the following comparisons at first: greater activation in PBD versus HR, in HR versus PBD, in PBD versus TD, in TD versus PBD, in HR versus TD, and in TD versus HR. However, two pairwise ALE meta-analyses (greater activation in PBD versus HR and greater activation in TD versus HR) were not performed due to the lack of available data. The input co-ordinates were weighted to form estimates of activation likelihood for each intracerebral voxel. The activation likelihood of each voxel in standard space was then combined to form a statistic map of the ALE score at each voxel. Statistical significance of the ALE scores was determined by a permutation test controlling the false discovery rate (FDR) at *p* < 0.05 (45). The statistic maps were thresholded by default at this critical value, and a recommended minimum cluster size was suggested from the cluster statistics. By using this minimum cluster size for the supra-threshold voxels, we can obtain the thresholded ALE image. Pairwise ALE analyses results were reported at *p* = 0.05 and were whole-brain corrected. A Talairach Daemon was used for anatomical locations for significant clusters.

## Results

**Figure 2:**
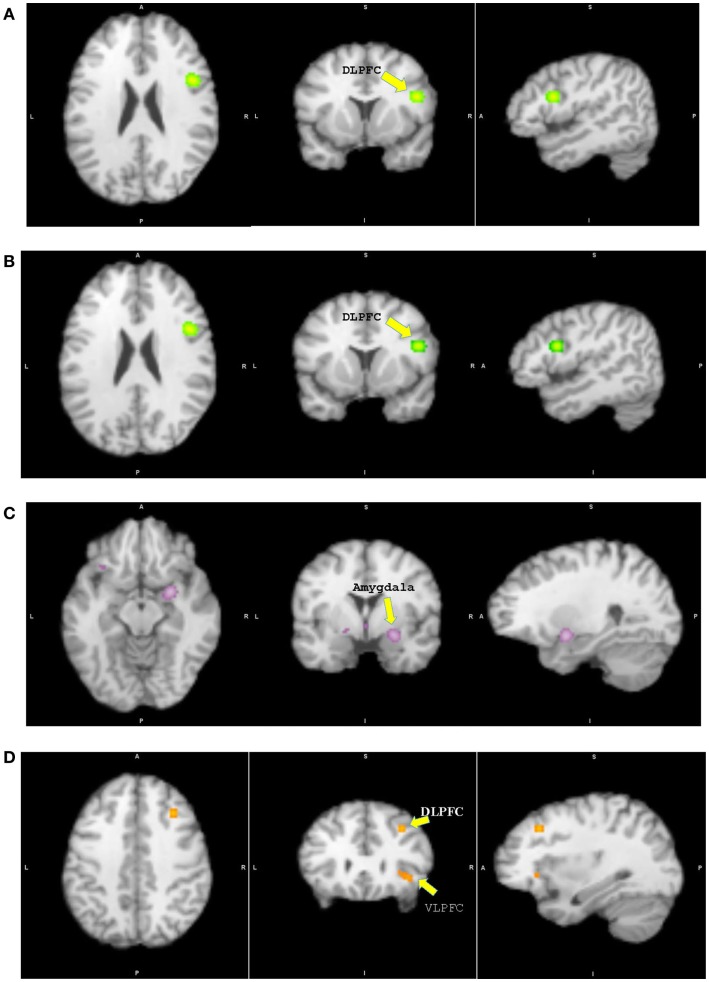
**Results from pairwise activation likelihood estimation (ALE) analysis**. **(A)** High-risk youth > typically developing youth. **(B)** High-risk youth > youth with bipolar disorder. **(C)** Youth with bipolar disorder > typically developing youth. **(D)** Typically developing youth > youth with bipolar disorder. **(A)** DLPFC: dorsolateral prefrontal cortex, *x* = 46, *y* = 8, *z* = 22, cluster size = 1048 mm^3^, extreme value = 0.020; **(B)** DLPFC: *x* = 46, *y* = 8, *z* = 22, cluster size = 1056 mm^3^, extreme value = 0.020; **(C)** Amygdala: *x* = 26, *y* = −2, *z* = −12, cluster size = 1120 mm^3^, extreme value = 0.022; **(D)** DLPFC: *x* = 32, *y* = 24, *z* = 38, cluster size = 224 mm^3^, extreme value = 0.013; VLPFC: ventrolateral prefrontal cortex.

The meta-analysis of all fMRI reports included 29 studies (PBD, HR, and TD). There was no overlap in patients who completed the same task across the selected studies. The primary studies included in the meta-analysis are listed in Table 1. Findings are summarized in Table 2 and Figure 2.

**Table 1:** **Primary fMRI studies of participants with pediatric bipolar disorder (PBD), those at high risk (HR) for PBD, and typically developing (TD): children included in meta-analysis**.

Primary study	Sample size	Age (mean ± standard deviation, years)	Medication status	Task
Cerullo et al. (24)	PBD (11, female = 7), TD (13, female = 6)	Age range: 11–18, PBD: 14.2 ± 1.5, TD: 14.5 ± 1.9	All bipolar participants had been off atypical anti-psychotics for at least 72 h and had undetectable levels of mood stabilizers.	Continuous performance task with a response inhibition component
Chang et al. (25)	PBD (12, all male), TD (10, all male)	Age range: 9–18, PBD: 14.7 ± 3.0, TD: 14.4 ± 3.2	All PBD participants except one were taking medication at the time of the fMRI.	Two-back visuospatial working memory task and an affective task showing emotionally valenced pictures
Deveney et al.^a^ (5)	PBD (19, female = 12), HR (13, female = 7), TD (21, female = 8)	PBD: 14.76 ± 2.9, HR: 13.46 ± 1.8, TD: 13.78 ± 2.0	10 of 19 PBD participants were medicated.	Stop signal task
Deveney et al. (26)	PBD (32, female = 17), TD (21, female = 8)	Age range: 8–18, PBD: 14.5 ± 2.5, TD: 13.8 ± 2.0	17 of 32 PBD youths were medicated.	Stop signal task
Dickstein et al. (27)	PBD (16, female = 7), TD (16, female = 7)	Age range: 7–18, PBD: 14.1 ± 2.5, TD: 13.9 ± 2.4	13 of 16 PBD youths were medicated.	Probabilistic reversal task
Dickstein et al. (28)	PBD (23, female = 14), TD (22, female = 12)	PBD: 14.2 ± 3.1, TD: 14.7 ± 2.3	18 of 23 PBD youths were medicated.	Encoding task and subsequent memory task
Diler et al. (29)	PBD (10, female = 8), TD (10, female = 8)	Age range: 12–17, PBD: 15.6 ± 0.9, TD: 15.6 ± 1.2	7 of 10 youths were medicated.	Emotional face gender-labeling task
Diler et al. (30)	PBD (10, female = 8), TD (10, female = 8)	Age range: 12–17, PBD: 15.6 ± 0.9, TD: 15.6 ± 1.2	All PBD youths were medicated.	Go/no go block design cognitive control task
Garrett et al. (20)	PBD (20, female = 6), TD (21, female = 8)	Age range: 9–17, PBD: 15.63 ± 2.10, TD: 15.35 ± 2.68	Exact total percentage of medicated participants was not shown.	Emotional (happy, sad, and neutral) facial expression
Kim et al.^a^ (6)	PBD (28, female = 16), HR (13, female = 7), TD (21, female = 8)	Age range: 8–17, PBD: 14.37 ± 2.63, HR: 13.90 ± 2.02, TD: 13.73 ± 1.96	18 of 28 PBD youths were medicated.	The change task
Kim et al. (31)	PBD (18, female = 8), TD (15, female = 10)	Age range: 9–18, PBD: 14.29 ± 2.54, TD: 14.98 ± 2.03	15 of 18 PBD youths were medicated.	Emotional face gender-labeling task
Ladouceur et al.^a^ (7)	HR (16, female = 7), TD (15, female = 11)	Age range: 8–17, HR: 14.2 ± 2.3, TD: 13.8 ± 2.7	All participants were unmedicated.	Emotional face N-Back task
Leibenluft et al. (32)	PBD (26, female = 14), TD (17, female = 8)	PBD: 13.6 ± 2.6, TD: 14.6 ± 1.8	13 of 26 PBD youths were medicated.	Stop signal task
Mourao-Miranda et al.^a^ (8)	HR (16, female = 9), TD (16, female = 9)	Age range: 12–17, HR: 14.8 ± 1.8, TD: 15.3 ± 1.2	All participants were unmedicated.	Emotional face gender-labeling task
Nelson et al. (33)	PBD (25, female = 13), TD (17, female = 8)	Age range: 8–17, PBD: 13.4 ± 2.5, TD: 14.6 ± 1.8	13 of 25 PBD youths were medicated.	The change task
Olsavsky et al.^a^ (9)	PBD (32, female = 15), HR (13, female = 6), TD (56, female = 30)	Age range: 8–18, PBD: 14.7 ± 2.7, HR: 14.0 ± 2.4, TD: 14.0 ± 2.6	24 of 32 PBD and 1 of HR youths were medicated.	Emotional face presentation
Passarotti et al. (34)	PBD (15, female = 8), TD (15, female = 8)	Age range: 10–18, PBD: 13.20 ± 2.65, TD: 14.13 ± 3.16	8 of 15 PBD youths had been medicated in the past. Patients were drug-free for at least 7 days before testing.	Stop signal task
Passarotti et al. (22)	PBD (23, female = 13), TD (19, female = 10)	Age range: 10–18, PBD: 13.55 ± 2.48, TD: 13.53 ± 3.16	All participants were medication free or had a washout period of at least 4–7 days before scanning.	Emotional face N-Back task
Passarotti et al. (35)	PBD (17, female = 11), TD (14, female = 7)	Age range: 10–18, PBD: 14.27 ± 1.98, TD: 14.14 ± 2.42	All participants were medication free or had a washout period.	Emotional valence Stroop task
Passarotti et al. (36)	PBD (17, female = 12), TD (13, female = 7)	Age range: 10–18, PBD: 14.29 ± 2.05, TD: 14.38 ± 3.57	All patients were medication free for at least 7 days prior to scanning.	Emotional face N-Back task
Pavuluri et al. (37)	PBD (10, female = 4), TD (10, female = 5)	Age range: 12–18, PBD: 14.9 ± 1.85, TD: 14.3 ± 2.36	All participants were unmedicated.	Emotional face presentation
Pavuluri et al. (21)	PBD (10, female = 5), TD (10, female = 5)	Age range: 12–18, PBD: 15.2 ± 2.0, TD: 14.3 ± 2.1	All participants were unmedicated.	Incidental and directed emotion-processing task
Pavuluri et al. (38)	PBD (13, female = 3), TD (13, female = 9)	Age range: 10–18, PBD: 14.4 ± 2.2, TD: 14.4 ± 2.8	All patients were medication free for at least 4–7 days prior to scanning.	Response inhibition task
Pavuluri et al. (39)	PBD (17, female = 11), TD (14, female = 7)	Age range: 12–18, PBD: 14.3 ± 1.1, TD: 14.1 ± 2.4	All participants were unmedicated.	Pediatric affective color matching task
Rich et al. (23)	PBD (22, female = 12), TD (21, female = 10)	Ager range: 9–17, PBD: 14.2 ± 3.1, TD: 14.5 ± 2.5	18 of 22 PBD youths were medicated.	Emotional face presentation
Singh et al. (40)	PBD (24, female = 11), TD (24, female = 15)	Age range: 13–18, PBD: 15.7 ± 1.7, TD: 15.0 ± 1.4	20 of 24 PBD participants had a history of medication exposure.	Monetary incentive delay task, affective priming task
Singh et al. (41)	PBD (26, female = 7), TD (22, female = 9)	Age range: 9–18, PBD: 15.4 ± 2.37, TD: 14.3 ± 2.33	History of medication exposure: valproic acid (13), lithium (8), antidepressants (16), atypical anti-psychotics (6), psychostimulants (14), or more than one medication (16).	Go/no go block design cognitive control task
Thermenos et al.^a^ (11)	HR (10, female = 5), TD (10, female = 5)	Age range: 13–24, HR: 18.4 ± 4.2, TD: 17.1 ± 1.4	All participants were unmedicated.	2-back working memory task and 0-back control task
Weathers et al. (42)	PBD (16, female = 8), TD (21, female = 9)	PBD: 14.65 ± 2.19, TD: 13.79 ± 1.97	9 of 16 PBD youths were medicated.	Stop signal task

*^a^Studies including HR groups*.

*Some studies were missing age range information and showed only the mean age. Accordingly, that information could not be included within the table. Specific medications were heterogeneous when reported and at times went unreported. Hence, we were only able to comment on participants’ medicated/unmedicated status. Similarly, the mood and affect of participants were also largely unreported and, therefore, could not be included in the table*.

**Table 2:** **Activation likelihood estimation (ALE) meta-analysis findings for fMRI studies comparing pediatric bipolar disorder (PBD) patients, participants with a high risk (HR) for PBD, and typically developing (TD) children**.

Pairwise analysis	Side	Brain region	BA	Talairach			Cluster size (mm^3^)	Extreme value
				*X*	*Y*	*Z*	
HR youths > TD youths (11 experiments)	L	Cerebellum, culmen		−8	−50	−26	1472	0.022
				−14	−36	−22		0.014
				−2	−54	−10	952	0.021
	R	Dorsolateral prefrontal cortex	9	46	8	22	1048	0.020
	R	Insular cortex	13	38	18	6	472	0.014
	R	Parietal lobe, inferior parietal lobule	40	32	−46	42	464	0.014
HR youths > BD youths (6 experiments)	R	Dorsolateral prefrontal cortex	9	46	8	22	1056	0.020
	L	Cerebellum		−8	−50	−26	944	0.022
	R	Insular cortex	13	38	18	6	496	0.014
BD > TD (43 experiments)	R	Amygdala, limbic lobe, parahippocampal gyrus,		26	−2	−12	1120	0.0221
	R	Frontal lobe, medial prefrontal cortex	10	4	62	14	872	0.030
				12	40	10	568	0.023
	L	Ventral striatum		−16	−12	28	640	0.024
	R	Somatosensory association cortex	7	42	−58	48	576	0.020
				2	−64	56	392	0.019
	L	Cerebellum		−16	−36	−24	560	0.022
	L	Lentiform nucleus, putamen, lateral globus pallidus		−22	6	−4	464	0.018
				−12	4	−6	368	0.017
				−16	−4	−8		0.013
	L	Ventrolateral prefrontal cortex	47	−30	20	−8	336	0.017
	R	Subgenual cingulate cortex	25	2	0	−4	256	0.016
TD > PBD (21 experiments)	R	Dorsal cingulate cortex	32	2	36	12	1576	0.017
	R	Dorsal striatum		10	10	6	696	0.014
	R	Ventrolateral prefrontal cortex	47	38	24	−4	336	0.011
	R	Dorsolateral prefrontal cortex	8	32	24	38	224	0.013
	R	Superior frontal gyrus	10	24	48	2	216	0.011

*R: right, L: left*.

### HR and TD: Recognizing high-risk participants

Participants in the HR group showed significantly greater activation in the right DLPFC, insula, inferior parietal lobule, and left cerebellum relative to TD. No other group differences were found. In case of greater activation in the TD group relative to HR, the analysis was not performed due to the lack of a large enough sample size and of experiments showing significant results.

### PBD and HR: Recognizing the emergence of the disorder

The HR group showed significant greater activation of the right DLPFC, insula, and left cerebellum than PBD. No other group differences were identified. In case of greater activation in the PBD group relative to HR, the analysis was also not performed due to a small sample size and few experiments showing significant results.

### PBD and TD: Recognizing the illness from wellness

Patients with PBD demonstrated greater activation in the subcortical regions of the right amygdala, the parahippocampal gyrus, the subgenual ACC, and the medial PFC, and in the left ventral striatum, VLPFC, and cerebellum relative to TD. The TD group showed greater activation in the right VLPFC, DLPFC, superior frontal gyrus, dorsal ACC, and striatum than patients with PBD.

## Discussion

We found the recently published developmental meta-analysis of bipolar disorder performed by Wegbreit et al. The researchers compared different age groups with bipolar disorder (youths and adults). PBD youths showed increased activation in the amygdala, the inferior frontal gyrus, and precuneus compared to bipolar disorder adults during tasks using emotional stimuli. These findings revealed that these structures are underdeveloped and work less efficiently when compared with those of adults (46). However, our meta-analysis was conducted using the comparison between participants of the same age (participants’ mean age is less than 19 years). The central findings of the meta-analyses of brain function among the PBD, HR, and TD groups, during the performance of mixed cognitive and emotional activities, illustrated a coherent pattern of group differences in line with our *a priori* hypothesis. The HR group showed a significantly greater activation in the *right DLPFC–insular–parietal–cerebellar regions* relative to TD, and this may be a bio-signature – an earlier sign of potential PBD development. At the junction of the DLPFC and VLPFC regions, where prefrontal systems interface in voluntary modulation of cognition, emotion, and motor control, brain function was amplified in the HR group (6, 7). Large future studies of symptomatic HR population (47) and genetic HR population must be compared both at a symptomatic and brain functional level to look at the definitive predictability of symptoms and the correlation of brain activity patterns.

A repeated and important observation of hemodynamics of the fMRI studies is the increased activity in the brain that reflects increased effort (48). If one construes TD as the reference point of normative activity, then the HR group showed increased effort to get the same work done by deploying the right DLPFC–insular–parietal regions relative to TD, while in PBD, these same regions went offline relative to TD. This finding is akin to the analogy of “stretching an elastic band” with increased DLPFC activity (requiring a greater effort than TD) in the HR group, whereas those with PBD who had a more severe illness had reached a breaking point with decreased right VLPFC and DLPFC activity (with no effort to spare relative to TD). We could not explain the increased left VLPFC activity in PBD relative to TD. While such a finding is not unexpected in a meta-analytic study, it was largely based upon the participants of only one study (21). However, it can be explained by bilateral disturbances in the VLPFC in PBD, albeit with the common and prominent right-sided abnormality than the left (32, 37). In the end, while one can postulate with explanations consistent with repeatedly published findings, definitive interpretations are not possible in understanding the nature of abnormal hemodynamic activity. For example, decreased (5) or increased (6) activation of the striatum with failed trials cannot easily differentiate HR from PBD based on any individual study. It could be mediated by the severity of illness in case of PBD, subsyndromal symptoms in HR, type of task, or hemodynamic relationship between the striatum and the PFC control regions.

With regard to recognizing the fully formed illness, typically noted underactivity of the higher cortical regions of emotion modulation (i.e., the interfacing dyad of the right VLPFC and DLPFC in the prefrontal regions) and overactivity of the subcortical amygdala consistently reported in BD Type I participants relative to TD adolescents (19) has also emerged as a significant finding in the current meta-analyses. The VLPFC is believed to serve the dual function of emotion (49) and motor (50) control via top–down regulation of the amygdala (51) and striatum (52), respectively. The DLPFC also serves a dual function, but it is predominantly through diverse cognitive functions involving executive control, response selection, problem solving, and emotion (53), and by being closely connected to the medial PFC, VLPFC, and the subcortical regions directly (54) as well as indirectly (52). The cognitive and emotion control regions in the PFC are not able to moderate the overactive subcortical regions, a consistent finding that was further underscored in our meta-analysis. In addition to the top–down *affect modulation circuitry* problems, increased activity is lateralized to the left side in the evaluative medial PFC, pregenual ACC, and the striatal loop (55); furthermore, all these regions are known to be closely connected to the amygdala (56). This subcortical and medial PFC loop is the *affective evaluation circuit* that is overactive in PBD. These findings could explain the excessive reactivity to negative emotions reported in PBD (21, 57) and are also in line with the concept suggested for bipolar disorder in general, including adult patients. Phillips and Swartz conceptualized bipolar disorder as multiple dysfunctions in prefrontal hippocampal–amygdala, emotion processing, and emotion-regulation circuits, together with an “overactive,” left-sided ventral striatal-ventrolateral, and orbitofrontal cortical reward-processing circuit (58). These results attest to the fact that, in relative terms of group comparison from fMRI studies, cognitive DLPFC and the corresponding dorsal circuitry hub that includes the parietal region and the insula are more involved in the HR population, while the wider multiple cortico (VLPFC, DLPFC, and medial PFC) and subcortical (limbic and basal ganglia) regions are implicated in PBD.

Published structural and fMRI studies of HR have not been conclusive and are limited to a comparison with the TD at times (7, 11). Singh et al. (59) reported that 8- to 12-year-old children with a familial risk for mania did not exhibit any statistically significant volumetric differences in the PFC, thalamus, striatum, or amygdala compared with the TD group. However, they concluded that longitudinal studies will be needed to examine whether structural changes over time may be associated with a HR for BD (59). Bechdolf et al. (60) reported volume reduction in emotion-processing regions (i.e., the insula and amygdala) in HR, relative to TD, that corresponded to the functional abnormality involving increased amygdala activity in HR (9). While we found abnormal function in the insula in HR in this meta-analysis, three-way comparison did not reveal increased amygdala activity in HR. Existing studies consistently reported smaller amygdala and hippocampus (61), larger basal ganglia (62), and reduced PFC gray matter (63) in PBD. Hemodynamic (64) and resting state connectivity (65) findings in PBD relative to TD also point to frontolimbic and frontostriatal functional disturbance in PBD. Such uniformity in multi-modal imaging findings attests to the high reliability in establishing a significant pattern of brain dysfunction specific to PBD.

Limitations of this study include fewer and unequal numbers of participants in the HR group and the inclusion of studies that employed variable tasks used to probe multiple domains. However, due to the broad array of daily functions that draws from the active involvement of multiple and highly integrated networks, and the dual engagement of VLPFC, DLPFC, ACC, and the striatum in both cognitive and emotional tasks, this study was a reasonable first attempt to examine the entire brain’s level of functionality from the existing data.

## Conflict of Interest Statement

The authors declare that the research was conducted in the absence of any commercial or financial relationships that could be construed as a potential conflict of interest.
